# A conceptual framework on body representations and their relevance for mental disorders

**DOI:** 10.3389/fpsyg.2023.1231640

**Published:** 2024-01-05

**Authors:** Anne Möllmann, Nina Heinrichs, Arvid Herwig

**Affiliations:** Department of Psychology, Faculty of Psychology and Sports Science, Bielefeld University, Bielefeld, Germany

**Keywords:** body representation, body image, body schema, developmental psychopathology, body dysmorphic disorder, muscle dysmorphia, eating disorders, body integrity dysphoria

## Abstract

Many mental disorders are accompanied by distortions in the way the own body is perceived and represented (e.g., eating disorders, body dysmorphic disorder including muscle dysmorphia, or body integrity dysphoria). We are interested in the way these distortions develop and aim at better understanding their role in mental health across the lifespan. For this purpose, we first propose a conceptual framework of body representation that defines this construct and integrates different perspectives (e.g., cognitive neuroscience, clinical psychology) on body representations. The framework consists of a structural and a process model of body representation emphasizing different goals: the *structural model* aims to support researchers from different disciplines to structure results from studies and help collectively accumulate knowledge about body representations and their role in mental disorders. The *process model* is reflecting the dynamics during the information processing of body-related stimuli. It aims to serve as a motor for (experimental) study development on how distorted body representations emerge and might be changed. Second, we use this framework to review the normative development of body representations as well as the development of mental disorders that relate to body representations with the aim to further clarify the potential transdiagnostic role of body representations.

## 1 Introduction

The way people perceive their own body, that is, represent it mentally, is essential in everyday life, and has thus been researched from a variety of disciplines and perspectives. Some researchers primarily focus on the *very basic functions* of body representations, such as being able to move in three-dimensional space which is usually a perspective taken in cognitive neurosciences, general psychology and motor sciences (e.g., Holmes and Spence, 2004; Prinz et al., 2013). Others concentrate on the normative *development* of different body representations over the lifespan (e.g., Slaughter and Brownell, 2011) which is usually a perspective taken in developmental psychology and again others are mainly interested in the role body representations play in the context of different *neurological or mental disorders*, a perspective usually taken in clinical psychology or psychiatry (e.g., Keizer and Engel, 2022 for anorexia nervosa). Due to the many perspectives involved, there is a certain diversity of concepts and terms. In this article, we therefore use the term body representation as an umbrella term for many aspects that have been differentiated to investigate the way the own body is mentally represented. Moreover, we attempt to elaborate on each perspective with the aim of illustrating their useful contributions ultimately to better understand mental disorders that relate to body representations.

Mental disorders associated with distortions in body representations are often severe and persistent. Such disorders include eating disorders, body dysmorphic disorder (BDD), muscle dysmorphia (MD), and body integrity dysphoria (BID). The distortions in body representation observable in the clinical picture of each disorder may relate either to the whole body or to specific body parts. The majority of the disorders are by criteria definitions (ICD-11; World Health Organization, 2018) rather related to one of these poles (e.g., criteria for eating disorders or muscle dysmorphia relate to the body perception as too fat or too lean/unmuscular). However, even with concerns relating to the entire body, it is not rare that also specific parts of the body are evaluated negatively. In BDD, these specific regions are often related to the face and perceived as defective or flawed, or in BID, affected individuals perceive single limbs as not belonging and thus desire an amputation (World Health Organization, 2018). On a general level, a shared feature of these disorders are distortions in the individuals’ body representation. However, it is challenging to integrate research findings within one disorder or to compare findings between disorders. This is due to conceptual and methodological aspects, such as a remarkable heterogeneity in terminology and assessments of concepts related to body representations. For example, we identified more than 120 different measures and tasks on body representations for a systematic review focusing on BDD, MD and BID only (Möllmann et al., 2020a). The problem is particularly evident in comparative research between disorders and/or disciplines, with each discipline usually preferring certain definitions of concepts and terms as well as different assessments (Smeets, 1997; Keizer and Engel, 2022). Importantly, these conceptual problems not only complicate the integration of findings, they also hinder improvement in understanding the disorders and related treatment options (see e.g., Gadsby, 2021 or Glashouwer et al., 2019 for a comprehensive discussion). Improving treatment options, in turn, is highly needed, as remission rates for most of the disorders associated with distorted body representations are rather low, e.g., between 20 and 39% in BDD (e.g., Phillips et al., 2013; Wilhelm et al., 2014; Mataix-Cols et al., 2015; Enander et al., 2016; Fernández de la Cruz et al., 2021) or around 50% in anorexia nervosa (Steinhausen, 2002).

As Gadsby (2017) emphasizes, the understanding about normative functioning of body representations can be improved by studying malfunctioning or distortions of body representations in respective disorders and vice versa. This underlines the importance to consider research from multiple perspectives or disciplines, such as cognitive neurosciences, and clinical psychology. In addition, we think it is very fruitful to also look at the interplay between normative and pathological *development* of body representations to further clarify if – and if so, how – these distorted body representations are involved in the development or course of the respective disorders. It is well-known that children face normative developmental challenges, for example in form of domain-specific tasks (such as learning to be prosocial) and that the (un-)successful completion is in turn associated with (impaired) further development in this domain with spreading effects to other domains as well (i.e., cascade effects; Masten and Cicchetti, 2010). It is important to consider the development of body representation as one such domain and we will address such developmental aspects in body representations below.

To better understand the role of body representations in mental health, we believe it is essential to provide a conceptual framework first. This framework should allow to (a) *integrate* the terminology and conceptual understanding of different perspectives and (b) *systematically review and investigate* body representations in normative development as well as in the development of psychopathology across disorders and disciplines. With this approach, we address several of the valuable recommendations from a comprehensive systematic review on body representations in anorexia nervosa (Glashouwer et al., 2019). To this end, we propose a framework consisting of two interconnected models, a structural and a process model.

In the present article, we *first* introduce the models in more detail, focusing on the structural model, which includes the integration of terminology and concepts from different perspectives. *Second*, we review the literature on the normative development of body representations within the proposed model, identifying potential sensitive developmental phases that may initiate cascade effects. *Third*, we review distortions in body representation from a transdiagnostic perspective across several mental disorders taking into account developmental aspects. In this sense, we follow a developmental psychopathology approach to mental disorders including distorted body representation. The specific role for body representations for these mental disorders is not always clear: while in some disorders certain aspects of body representations may be assumed to be a risk factor (e.g., anorexia nervosa), for others there has been less research and a lack of experimental and longitudinal studies that would help clarifying if distorted body representations are a feature of the disorder, an epiphenomenon or a (causal) risk factor. The development of the models we suggest in the next section is supposed to help clarifying this important question.

## 2 Conceptual framework on body representations: a structural and a process model

**Figure 1:**
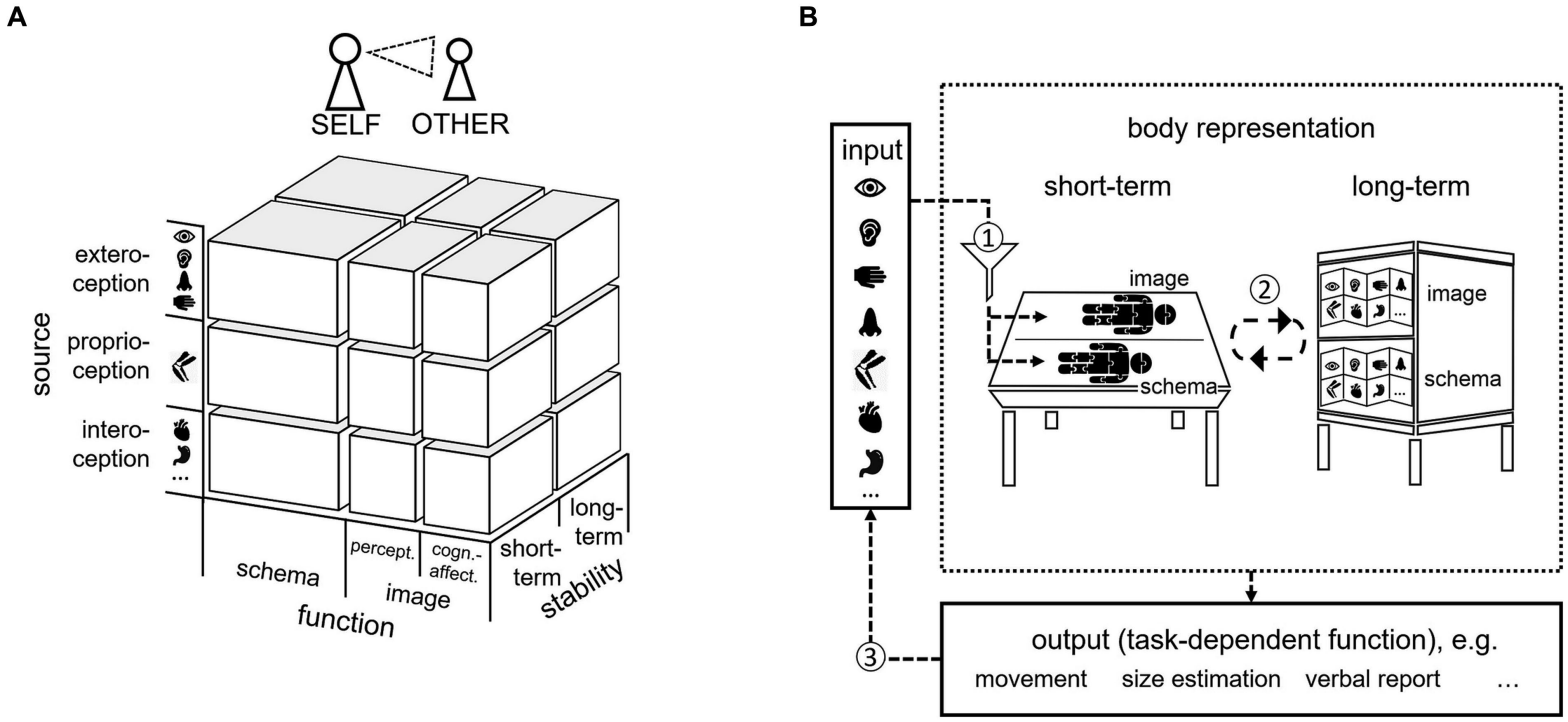
Conceptual framework of body representations consisting of **(A)** a three-dimensional structural model of body representation and **(B)** a process model of body representation. The structural model **(A)** considers three dimensions (function, source, stability; see text for further details) on which body representations can be differentiated. Moreover, the structural model can be used to classify representations of ones’ own body as well as representations of the bodies of others (self-other). The process model **(B)** depicts the dynamics during the information processing of body-related stimuli. Accordingly, a short-term representation emerges from (1) weighted sensory information about the body as it currently is from different sensory sources (exteroceptive, proprioceptive, and interoceptive) and (2) stable information stored in long-term representations about the body as it usually is. Short- and long-term representations are combined in the service of functions (e.g., movement, size-estimation, verbal report) which might lead to (3) behavior-induced modifications of sensory input.

Our conceptual framework on body representation includes a structural model (categorial approach) and a process model (dynamic approach). Both serve different purposes as outlined below. In developing this framework, we integrated and extended conceptualizations, models, and empirical findings of researchers from different disciplines, such as cognitive (neuro)sciences, philosophy, experimental and clinical psychology (e.g., De Vignemont, 2010; Cash and Smolak, 2011; Longo, 2016; Vocks et al., 2018; Gadsby, 2021). The *structural model* (Figure 1A) may be especially *useful to categorize* different types of body representations and thus to *systematize* previous research or develop targeted new research questions – both from different research perspectives as well as for different mental (or other) disorders. Further, it may be used regarding both, the representation of one’s own body and the representation of others. In contrast, the *process model* focuses on the (situational) development and usage of a body representation. It thus rather reflects a dynamic information processing model on body representation (Figure 1B), illustrating how a body representation emerges in the short- and long-term. As Medina (2022) pointed out, this kind of process model is highly needed in addition to more categorical approaches for the development in the field of body representation research.

In the present article, we will primarily focus on the structural model as it is our goal to first demonstrate how the presently available results from studies on body representations may be structured to identify and accumulate knowledge available and to inform future research (areas). We will re-visit the process model in the discussion section again to demonstrate how it may be used once the evidence has been successfully structured.

### 2.1 The three-dimensional structural model of body representations

As visualized in Figure 1A, the structural model considers three dimensions on which body representations can be differentiated:

*Functions of body representations*: body schema vs. body image (perceptual and cognitive-affective).

*Sensory source*: exteroception, proprioception and interoception.

*Stability:* short-term vs. long-term.

Importantly, the model suggests differentiating between body image and body schema in contrast to synonymously use the terms, and it suggests separating body representations regarding the primary source of information. Moreover, it distinguishes between body representations that are short-term representing the body as it currently is and long-term representing the body as it usually is. Finally, the structural model can be used to classify representations of ones’ own body as well as representations of the bodies of others (*self-other*). We will outline each dimension in more detail in the following sections.

#### 2.1.1 Function of body representation: body schema vs. body image

We named the first dimension of the structural model *function* to underline our understanding that each body representation is needed and used for a specific purpose, a function, such as to execute a movement, perform a size estimation or answer questions about one’s body (see Figure 1B, Process model, output). In clinical psychology, unlike in other disciplines, the term body representation as an umbrella term is rather uncommon. Instead, researchers are usually familiar with a certain understanding about the body image, a concept that is often used and understood synonymously to the body schema (or perceptual body image and body schema as synonyms). The synonymous use of the terms body image and body schema is unusual in other disciplines, such as neuroscience, where the terms characterize different functions of body representations. As this has led to challenges in integrating research findings (Glashouwer et al., 2019), we suggest, to align terminology across disciplines under the in some areas already established umbrella term body representations and use specific subterms for respective functions (i.e., body image and body schema) or other aspects of body representations.

In the structural model, we thus differentiate *on a first level* body image and body schema and subdivide body image further into the cognitive-affective versus perceptual body image *on a second level*. We define the cognitive-affective body image as feelings and attitudes toward one’s body, as in common models on body image (e.g., Gallagher, 1986; Cash and Smolak, 2011; Vocks et al., 2018). It includes body (dis)satisfaction (i.e., the evaluation of the physical appearance) and the importance of the appearance for someone’s self-evaluation. We define the perceptual body image as the representation of body “estimates,” typically regarding size and shape (c.f. body percept, Longo, 2016; Gadsby, 2017) but also regarding the body composition related to other sensory sources, e.g., the intensity of bodily sounds or odors (see next section). In contrast to some other models on body image (e.g., Cash and Smolak, 2011; Vocks et al., 2018), we did not include a behavioral component, referring to body-related behaviors, such as body-checking in eating disorder. As we certainly agree that these behaviors are important in many regards, we would argue that they are not body representations in themselves but rather are informed by or result from (distorted) body representations – and also inform or change body representations (see process model). The body image, in distinction to the body schema, is not action-oriented but the subjective experience of one’s body (c.f., Longo, 2016).

In contrast, we define the body schema according to De Vignemont (2010) as a function of body representation that is necessary for body-scaled action and/or motor imagery (Gadsby, 2017). It enables spontaneous, speeded movements, action or action simulation while considering the dimensions of the own body and the environment. The body schema develops bottom-up, without higher cognitive processes such as feelings or attitudes toward one’s appearance, and it includes representations of the body both as the effector and as the goal of the action. Differentiating functions of body representations does not mean that the functions are unrelated. Interactions between different aspects seem very likely.

The conceptualization of body image and body schema as mentioned above differs from the common terminology in clinical psychology. However, rethinking and specifying the current understanding of body image might contribute to efforts of integrating previous research (Glashouwer et al., 2019). Accordingly, we believe that differentiating body image and body schema can improve the understanding of the way body representations are distorted in mental disorders, such as eating disorders, BDD, MD, and BID, and eventually improve treatment options. For example, such a differentiation led to important research findings in anorexia nervosa. Engel and Keizer (2017) showed that after receiving cognitive-behavioral therapy (CBT), individuals with anorexia nervosa still revealed a distortion of the body schema. As CBT usually focuses on the cognitive-affective component of body image, these findings motivated the authors to develop intervention approaches that focus specifically on other functions of body representations. A similar approach would be important for under-researched disorders, such as BDD, MD, and BID as well.

#### 2.1.2 Sensory sources: exteroception, proprioception and interoception

We receive a continuous flow of sensory information about the body from various sources. These sources can be roughly parceled into exteroception, proprioception and interoception (Ceunen et al., 2016; Khalsa et al., 2018). Exteroception refers to the perception of the body and the environment from the outside. This includes sensory information that stems from the sensory organs connecting the outside environment to our inside via signal transmission, such as the skin, eyes, ears and nose. Proprioception, on the other hand, refers to the perception of the position and movement of limbs and the body as a whole. This includes information about muscle contractions, joint angles and the position of the body in space. Finally, interoception refers to the perception of stimuli and information from inside the body, such as the physiological states of the organs, digestion, or heart rate. Intertwined with this proposed tripartite division is the somatosensory system (De Haan and Dijkerman, 2020). In addition to exteroceptive tactile perception, the somatosensory system includes proprioception and interoceptive sensations of pain and temperature. Here, we adhere to the initial categorization of sensory sources (i.e., exteroception, proprioception, and interoception), as it provides a more comprehensive picture of sensory sources while still allowing for insights into the somatosensory system.

While proprioception and some prominent exteroceptive modalities (i.e., visual and tactile) are often taken into account in research and it may therefore be easy to understand why they are part of the model, the incorporation of interoception as well as further exteroceptive modalities (e.g., auditory and olfactory) might not be so obvious at a first glance. However, interoception has been increasingly recognized as a significant factor in body representations and various mental health conditions including eating disorders (e.g., Badoud and Tsakiris, 2017; Khalsa et al., 2018; Raimo et al., 2021). Moreover, the sounds and odors emanating from our bodies are a further exteroceptive source that affects the way the own body is represented. For example, sounds (e.g., produced when breathing, eating or walking) have an effect on the overall body awareness (Murray et al., 2000; Azañón et al., 2016) and can even alter one’s own perceived body weight (Tajadura-Jiménez et al., 2015a) as well as the mental representation of arm length (Tajadura-Jiménez et al., 2012, 2015b). Likewise, scent stimuli can affect participants’ perception of body shape and weight (Brianza et al., 2019) and booster the embodiment of a sex mismatching body in virtual reality (Lesur et al., 2023). This research on the significance of certain sensory sources for body representations, such as auditory and olfactory information, is still in its infancy, as these sources were more difficult to control and record experimentally in the past. However, we think that a comprehensive model on body representation should consider all information available about one’s own body. This evaluation is also supported by clinical conditions, such as misophonia and the olfactory reference disorder, where (the processing of) this sensory information is closely related to the disorder symptoms. Misophonia describes a reduced tolerance toward certain sounds or associated stimuli, most often related to eating or breathing sounds from individuals close to the affected person, such as a partner or parent (Swedo et al., 2022). Individuals with misophonia thus develop very detailed body representations regarding other people’s bodily sounds. In olfactory reference disorder, individuals are preoccupied about emitting a strong body odor which is not or only slightly noticeable for others (Schmidt et al., 2021). This characteristic affects the body image, based on olfactory information.

Importantly, information from different sensory sources are often encoded in separate brain regions and separate spatial frames of reference. For example there are over a dozen distinct maps encoding visual and tactile information about the body spanning the parietal and frontal cortex (Huang et al., 2012) as well as a multitude of body-sensing brain regions including the insula and somatosensory cortices which form the neural basis of interoception (Paulus et al., 2019). This requires elaborate computations to combine or integrate these different inputs (Ernst and Bülthoff, 2004; Kirsch and Kunde, 2023). The exploration of the neural pathways and processes underlying these computations is constantly expanding, which is not surprising given the multitude of sensory sources and their potential interactions. Therefore, an in-depth review of the neuronal basis of sensory integration is beyond the scope of this article and can be found elsewhere (e.g., Murray and Wallace, 2012; Yau et al., 2015). From this research, it can be noted that multisensory integration occurs within a broad hierarchically organized neural network that involves numerous feedback loops. Of particular relevance in this network is the parietal cortex which is involved in reference frame remapping and mediating crossmodal interactions (e.g., between vision and touch) in sensory cortex (Yau et al., 2015). Moreover, behavioral studies on multisensory integration have provided evidence that integration is task-dependently taking into account, for example, the relative reliabilities of the sensory input (Ernst and Banks, 2002). As a consequence, different sensory sources are used in the formation of body representations (De Vignemont, 2010; Peviani et al., 2019) depending on the task, reliability, and availability of sensory information. Although body-related distortions may be the result of faulty multisensory integration (e.g., Saetta et al., 2020), distortions often occur at an earlier stage (i.e., before integration) and thus originate at the level of individual sensory sources (e.g., Feusner et al., 2007; Keizer et al., 2011; Longo, 2022). Identifying where and how exactly the processing of sensory information about the body is disturbed in mental disorders is challenging given the intricate recurrent processing loops underlying uni- and multisensory processing. However, we believe it is important to underline the relevance of the *main* constituting sensory source for a specific body representation. Thus, we suggest body representations can (and should) be further differentiated according to their main constituting sensory source. Accordingly, the sensory sources serve as a distinct, orthogonal dimension in our structural model of body representations.

Referring to the other two dimensions of function (body image vs. body schema) and stability (short-term vs. long-term; see next section), some sources appear to be more central than others. For example, proprioceptive information is especially relevant for the body schema due to its close relationship to movement. However, actions can also be selected and controlled based on exteroceptive information (e.g., auditory, visual, or olfactory information Herwig et al., 2007; Smeets and Dijksterhuis, 2014; Herwig, 2015). Moreover, research on the rare case of deafferented patients lacking proprioceptive input suggests that these patients’ body schema can instead be based on exteroceptive visual input only (De Vignemont, 2010). Likewise, interoceptive signals have been linked to motor control and the subjective experience of agency (Pezzulo et al., 2015; Marshall et al., 2018). Especially interoceptive signals related to itch and pain play an important role in actions aimed at the own body (Berlucchi and Aglioti, 2010). In case of body image, which is for example used for the perception of body size, exteroception (and especially visual information) seems to be more central than other modalities. However, the body image can also be based on other sensory information when visual input is not reliable or available (e.g., to estimate the size and shape of the back of one’s head) and is known to be affected by tactile sensitivity (Linkenauger et al., 2015), as well as bodily sounds and odors (e.g., Tajadura-Jiménez et al., 2015b; Lesur et al., 2023). Disturbed interoceptive information also alters the body image as evident by effects of local anesthesia, pain and cutaneous stimulation on the perceived size of body parts like the thumb and lips (Gandevia and Phegan, 1999).

In the case of mental disorders, some symptoms are more related to specific sensory sources than others. For example, body checking as a frequent symptom of eating disorders or BDD mostly leads to visual or tactile information which might render body representations. On the other hand, altered visual and proprioceptive information seems to be more relevant when it comes to pretending behavior (e.g., using crushes or wheelchairs) as one symptom of BID.

#### 2.1.3 Stability: short-term vs. long-term

Body representations can be further classified according to their temporal stability in short-term and long-term body representations (Merleau-Ponty, 1962; O’Shaughnessy, 1980; Carruthers, 2008). Short-term or online body representations are dynamic representations of the body as it currently is. As such they are constantly updated in working memory by currently available sensory information (e.g., by a look into the mirror, or by proprioceptive information about the current body posture). In contrast, long-term or offline body representations are more stable and represent the body as it is usually like. Typical properties of long-term body representations are the spatial organization and the size of body parts as well as learned movement possibilities or generalized motor programs (Berlucchi and Aglioti, 1997, 2010; Shea and Wulf, 2005; Schack and Mechsner, 2006).

As elaborated by De Vignemont (2010), the stability dimension (short-term vs. long-term) is orthogonal to the perception-action (body image vs. body schema) distinction. Moving the own body typically requires an interaction of both, short-term and long-term body schema representations. That is, for an arm movement, short-term information of the current position needs to be combined with long-term information about its size and learned movement possibilities (De Vignemont, 2010). The same holds true for body perception, which can be also conceived as an interaction between short-term and long-term body image representations.

It has been suggested that disturbances in body representation, as reported for eating disorders, are more likely due to disturbances in long-term representations permitting discrepant sensory input (Gadsby, 2017). Accordingly, dynamic short-term representations are thought to be corrupted by distortions imported from long-term representations. In fact, studies on anorexia nervosa indicate that disturbances might result from a biased weighting of short- and long-term information about the own body (Case et al., 2012; Keizer et al., 2013; Gadsby, 2017). For example, an oversized body representation of patients suffering from anorexia nervosa might be the result of a failed updating of long-term representations after sudden weight loss or of a general devaluation of current sensory short-term representations (Guardia et al., 2012; Gadsby, 2017). Likewise, it has been suggested that a failure to integrate short-term and long-term body representations leads to BID (Carruthers, 2008). It has to be noted, however, that interactions between long-term and short-term body representations are manifold. On the one hand, long-term body representations affect short-term representations as information from long-term memory is typically combined with the actual sensory input in working memory. On the other hand, short-term representations affect long-term representations as long-term information itself is constructed by accumulating short-term information about the own body. Thus, this dimension might be especially interesting from a developmental perspective on body representation and due to its dynamic nature studied in the context of our process model.

We will now move on focusing on the normative development of body representation, beginning with the “function” dimension of the structural model, body schema and body image, from a developmental perspective. We will refer to the source dimension alongside, differentiating the different senses where applicable. The stability dimension of the structural model will not be reviewed here from a developmental perspective as we believe the process model to be more suitable to investigate this dimension.

## 3 The normative development of body representations

How and when do different body representations emerge and develop during ontogenesis? In the following, we will first address this question in the course of normative development. This will help to better identify deviations and disturbances in the development of body representations that may accompany certain mental disorders as discussed later. Following the structural model of body representations outlined before, we will address these questions separately for body schema, and body image (including the perceptual body image and the cognitive-affective body image).

### 3.1 Body schema

The existence of a body schema, that is the body representation supporting body-scaled actions, can be found from very early on. An important discovery was the neonate’s ability to imitate facial gestures, requiring some sort of mapping between vision and motor response (e.g., Meltzoff and Moore, 1994; Meltzoff et al., 2013). Moreover, three to 5 months old infants already discriminate between pre-recorded and live videos of own leg movements (Bahrick and Watson, 1985; Rochat and Morgan, 1995), thus showing early integration of visual and proprioceptive signals during movement execution. This has led to the suggestion that certain parts of the body schema (e.g., supporting early manual-oral behaviors) are innate (e.g., Gallagher, 2005; Rochat, 2010), thus providing the foundation for the later developing body image (Brownell et al., 2012; see the discussion of the body image in the next section). However, other authors view the body schema as the result of pre- or postnatal experience (Merleau-Ponty, 1962; see also Bremner, 2017). Innate or not, there is evidence that the body schema is highly plastic and further develops during infancy and childhood, reaching maturation not until adulthood.

For instance, studies indicate that premature birth might impact on the later development of the body schema and associated motor skills as well as sensorimotor functioning (De Kieviet et al., 2009; Di Rosa et al., 2016; Butti et al., 2020). One of the reasons for these effects might be that preterm infants are exposed to atypical handling routines and thus experience atypical sensory stimulation (Peng et al., 2009). Interestingly, preterm children at the age of 8 to 14 years were less efficient than full-term children in expressing hand-laterality judgments on whole body stimuli (Butti et al., 2020). This finding provides a first tentative hint that early body-related experiences during the first year of life might initiate a developmental cascade impacting children’s developmental course. However, these difficulties were present regardless of whether the body was shown from the front (and thus requiring a first-person mental transformation that is known to involve one’s own body schema) or back (not necessarily involving the body schema). Further studies are needed to pinpoint the specific impact of premature birth on the body schema.

A further line of evidence for developmental effects on the body schema comes from tool use. For instance, children start using tools like rakes to reach a desired toy during the second year of life (e.g., Rat-Fischer et al., 2012). In adults, tool use modifies the body schema as indexed by a change in the kinematics of subsequent free-hand movements following a period of tool-use actions. More precisely, after using a mechanical grabber to pick up objects, adults move their arm as if it has become longer (Martel et al., 2019). Thus, tools become incorporated into the body schema and this incorporation lingers on for a certain amount of time after the tool is no longer in use. Importantly, the plastic capability of updating the body schema is strongly affected by the pubertal status of 8–21 years old (Martel et al., 2021). During early puberty, the kinematic changes that linger after a period of tool-use oppose the patterns observed for adults - children move their arm as if it has become shorter. This pattern reverses after mid-puberty with the typical adult-like pattern emerging only at late puberty, when body size stabilizes. Comparable shaped developmental trajectories have also been observed for localisation performance of the hand during movements (Nardini et al., 2013), kinaesthetic acuity (Visser et al., 1998), as well as the use of proprioceptive information for postural control of the upper limbs (Viel et al., 2009). Together, these findings point to the beginning of puberty as a further sensitive stage in the development and maturation of the body schema. After a period of constant growth during childhood, the rate of body growth increases sharply during the growth spurt leading to critical and fast changes in body morphology. This might present special challenges to the developing body schema and may therefore also be considered a vulnerable phase for body representation distortions due to the comprehensive adaptions in mental representation of the body (Assaiante et al., 2014; but see De Haan et al., 2018).

In sum, the findings on the development of the body schema implicate that although the body schema begins to develop already during a prenatal stage (or might be even partly innate), it remains highly plastic until adulthood and is therefore vulnerable for abnormal developments at least until then. Premature birth is a possible risk factor for the later development of such abnormalities, and puberty appears as a particularly sensitive phase due to the comprehensive changes in body morphology.

### 3.2 Body image

#### 3.2.1 Perceptual body image

As concerns the perceptual body image, i.e., representations of any body estimates (e.g., the body’s shape, size, spatial configuration, intensity of bodily sounds or odors), there is converging evidence that the abilities to represent our own bodies take significant developmental time to emerge (e.g., Brownell et al., 2012; Bremner, 2022). Especially during the first 3 years of life, different aspects of the perceptual body image emerge and develop at different points in time. For example, the ability to locate touch changes in children between 4 and 6 months of age, as localization performance becomes increasingly influenced by body shape and configuration. At the age of 4 month, children locate touch, as indexed by a tactile orienting response, equally well on their crossed and uncrossed feet. At the age of 6 month, however, children showed a tactile localization deficit with their feet crossed, indicating external spatial coding of touch (Begum Ali et al., 2015). This deficit to locate touch on crossed limbs is also apparent in sighted (but not blind) adults and arises from the influence of representations of the canonical posture of the body in external space (Röder et al., 2004; Badde et al., 2019). Body representations underlying the ability to locate touch are thus strongly affected by visual input during the first months of life.

Children in the first half of the second year initially become aware of individual body parts in isolation from one another. At this age, they can point to own body parts in response to a verbal label (i.e., auditory input) but have difficulties to match someone else’s body parts to their own using visuo-spatial information alone (Brownell et al., 2012). Between 18 and 22 months, children become first aware that their bodies, like other objects, take up space and can serve as obstacles, which is for instance demonstrated by a decline in attempts to hand over a mat they are sitting on without first moving off it. Body size awareness emerges a little later between 22 and 26 months, as indexed by fewer scale errors like trying to put on doll clothes or to squeeze through a too-small opening (Brownell et al., 2007). Only by the age of 30 months, children start to represent their bodies in terms of a more detailed topographic representation and are now able to correctly place stickers on unnamed body locations on themselves using visuo-spatial information as well as imitate meaningless gestures positioned at different body locations (Brownell et al., 2010).

The perceptual body image develops further in childhood as indicated by studies investigating the rubber hand illusion (Botvinick and Cohen, 1998). In this illusion, the sight of a fake hand being synchronously stroked with the real hand hidden from view, causes a shift in the localization of the real hand. More precisely, the real hand is perceived to be closer located to the seen fake hand after synchronous compared to asynchronous stroking. This self-localization without the possibility to move the real hand should be mainly based on the perceptual body image using visual, tactile and proprioceptive information. While children between 4 and 9 years are as sensitive as adults to visual-tactile synchrony cues for hand position, they perceive their hand generally (i.e., regardless of synchrony) to be located closer to the fake hand than 10 to 13 years olds as well as adult’s (Cowie et al., 2013, 2016). This might indicate two processes in the development of the perceptual body image during childhood. One process is based on the perceived temporal synchrony between visual and tactile stimulation which matures by at least 4 years of age and changes little after that. The other process is based on the integration of visual and proprioceptive information and matures not until 10 years of age. The latter finding fits well to the idea that children, at least before they enter puberty, rely more strongly on their visual than proprioceptive body image for self-localization. One possible reason might be that children’s proprioception needs more developmental time than vision to provide reliable estimates.

This finding is further complemented by a study investigating the spatial and temporal rules for multisensory integration underlying body representations in a sample of 5 to 12 years old (Greenfield et al., 2017). In this study, children had to report whether they saw their visually displaced hand in the same position as their actual hand and whether they experienced a tactile stroke to their hand as synchronous with a temporal delayed video of their own hand. Younger children showed an increased probability to mistakenly integrated together body related information from different modalities separated in space or time. Such broader integration windows of multisensory information might also partly underly the finding of younger children’s tendency to locate their real hand closer to the fake hand even after asynchronous brushing reported by Cowie et al. (2013, 2016).

In sum, the perceptual body image develops as the infant’s and child’s information about its body becomes richer and more reliable. A large part of the foundations is laid in early infancy and childhood, when visual information becomes incorporated in representations used to locate touch and body awareness slowly builds up. As the child growths and reaches puberty, the visual dominance in self localization decreases and integration windows of multisensory information narrow.

#### 3.2.2 Cognitive-affective body image

Research on the development of the cognitive-affective body image during childhood and adolescence appears more strongly directed toward distortions (e.g., body image concerns or body dissatisfaction) rather than toward the normative development of attitudes, beliefs or feelings about one’s body (Smolak, 2011; Paxton and Damiano, 2017). In comparison to the body schema and the perceptual body image, the cognitive-affective body image develops later, as the evaluation of one’s own body requires certain self-reflective cognitive functions.

One hypothesis is that it starts developing around the age of five, as comparisons with others become more important in this phase (Smolak, 2011). This hypothesis is derived from a common assumption, that the cognitive-affective body image in relation to oneself may be closely associated with the cognitive-affective body image in relation to others (see also the self-other aspect in our structural model, Figure 1A), e.g., in the weight bias. This bias describes negatively biased assumptions about others, for example regarding their intelligence, kindness or competences, based on their (higher) body weight (Puhl and Suh, 2015; Spiel et al., 2016). It is assumed that these negative attributions are initially only applied regarding others before they are also applied in relation to oneself and may thus lead to body dissatisfaction (Durso and Latner, 2008; Latner et al., 2013). Overall, however, the differentiation self - others appears especially important during childhood and adolescence across the development of different body representations.

Deviating from the hypothesis, other studies indicate that the cognitive-affective body image already starts developing earlier. Body (dis)satisfaction and weight bias are researched in children from about 3 years of age (Spiel et al., 2012), commonly using the current-ideal discrepancy score from figure rating scales, in which the child is presented with a certain number of body silhouettes that differ in terms of body weight (e.g., Damiano et al., 2015a,b). The children are then asked to select their current self on the one hand and their desired/ideal self on the other. In the case of agreement, this is evaluated as body satisfaction, in the case of deviation as dissatisfaction. These studies show that children from the age of around three are developmentally able to make evaluative judgments about their bodies. Further, a sensitive developmental phase for the body-related self-evaluation around the age of six to seven might be concluded from the studies with a kind of crossing point regarding body dissatisfaction (Dohnt and Tiggemann, 2006). Boys and girls at 4 years show very small discrepancy scores with 72 and 62% being satisfied with their bodies and within the groups of dissatisfied children, twice to three times as many desired a larger figure (Damiano et al., 2015a). With increasing age, the proportion of satisfied children begins to decrease, while the thinner ideal gains in importance and from age 6 there is a rapid increase in dissatisfaction with consistent findings of up to half of the children up to age 12 desiring a thinner ideal (Schur et al., 2000; Truby and Paxton, 2002, 2008; Ricciardelli et al., 2009).

A positive cognitive-affective body image has been repeatedly linked to higher interoceptive acuity (e.g., in a heartbeat perception task) in adults (Duschek et al., 2015; Emanuelsen et al., 2015; Badoud and Tsakiris, 2017; Todd et al., 2019). Thus, taking a developmental perspective on interoception (Murphy et al., 2017) might indirectly inform about critical phases in the development of the cognitive-affective body image. There is very little data on interoception in early infancy and it is not exactly clear at which age explicit awareness of interoceptive signals develops. However, implicit interoception using cortical measurements of cardiac information has been investigated in children aged 3 years and older (Immanuel et al., 2014). In this study, children with sleep disordered breathing showed reduced implicit interoception which correlated with problematic daytime behavior. The first explicit measurements of interoceptive sensitivity have been obtained in children aged 6 years and older (Koch and Pollatos, 2014). Here, low explicit interoceptive sensitivity (i.e., heartbeat perception) has been linked to both obesity and the presence of disordered eating behaviors over a one-year timeframe. Likewise, reduced explicit interoceptive sensitivity is associated with adolescence obesity in a sample of 12- to 18-year-olds (Mata et al., 2015). Although data on the normative development of interoception is not abundant, it has been suggested that interoceptive ability varies throughout development and is associated with periods of risk for the development of psychopathology (Murphy et al., 2017). Future studies should thus trace the link between interoception and body representations across different developmental periods.

### 3.3 Significance of developmental processes for distorted body representation

In summary, the different functions of body representations, body schema, perceptual body image and cognitive-affective body image, start developing at different ages with the earliest development in body schema, followed by the perceptual body image and cognitive-affective body image. They reveal a different level of plasticity and therefore susceptibility for the development of distortions, again during different phases of development. Regarding the sensory sources, most studies focus on exteroception (mainly visual, tactile) and proprioception. Studies on the normative development of interoception are rare but could play a greater role in the future, as atypical interoception could be closely related to the development of psychopathology. The role of other exteroceptive sources (i.e., auditory and olfactory) in the development of body representations is mostly neglected. Whether (and if so, how) interferences during the development of a specific body representation lead to a distorted body representation and affect the subsequent development of other representations, might be an important question for future research. From a clinical perspective, these phases of increased plasticity and therefore susceptibility for the development of distortions might be a gateway for the development of mental disorders characterized by distortions of body representation.

## 4 Mental disorders and distorted body representations

In this section we will now turn to apply our model to a selection of mental disorders and examine if distorted body representations are a transdiagnostic relevant factor for mental disorders. While a more comprehensive systematic review on existing studies would be necessary for answering such a question, and specifically more longitudinal as well as experimental studies, we aim to start at this point with a brief and exemplar review of studies. We will first shortly describe the clinical features of mental disorders typically associated with distorted body representations. Second, following the structural model of body representations, we will review each function, body schema and body image (perceptual and cognitive-affective body image), regarding research findings on distortions present in the respective mental disorders. Third, we will synthesize these findings in relation to the applicability of our model.

Several mental disorders are *typically* associated with distorted body representations, such as eating disorders, body dysmorphic disorder (BDD) including muscle dysmorphia (MD), and body integrity dysphoria (BID). It may be noted that beside these *typical* disorders, body representations as conceptualized in our framework may appear relevant for other (mental, neurodevelopmental) disorders or conditions as well, for example the olfactory reference disorder, pain disorders, misophonia, autism, or gender incongruence. For the scope of the current article, we will limit this section to eating disorders, BDD, MD and BID to apply the model for the first time.

### 4.1 Key features of mental disorders typically associated with body representation distortions

Regarding the key features of the disorders, eating disorders, such as anorexia nervosa (AN), bulimia nervosa (BN), and binge eating disorder (BED), are characterized by the preoccupation with one’s body weight and shape (ICD-11; World Health Organization, 2018) with slight differences between disorders. For example, a disturbed way of *perceiving* one’s weight or body shape is especially emphasized for AN (DSM-5-TR; American Psychiatric Association, 2022), while the overly importance of weight and shape for *self-evaluation* can be found across eating disorders (Grilo et al., 2019; Lydecker et al., 2022). In BDD, a disorder classified in the obsessive-compulsive spectrum, individuals are preoccupied with at least one aspect of their physical appearance which they perceive as flawed, defective, or deformed (American Psychiatric Association, 2022). They evaluate this or these aspect(s) of their appearance as unattractive or even ugly and hideous (Veale and Neziroglu, 2010). The perceived flaws are not at all or only slightly visible for others and up to 53% of individuals with BDD even experience delusional beliefs regarding their perceived defect (Phillips, 2004). MD, as a form of BDD, describes the specific preoccupation with one’s perceived insufficient muscularity (Cunningham et al., 2017). Several authors see MD more closely related to anorexia nervosa than to BDD, as the main concern similarly involves the shape of the whole body and is weight-related (Pope et al., 1993; Phillipou et al., 2016). BID is different from eating disorders, BDD and MD on the phenomenological level. It describes individuals with the intense and persistent desire to become physically disabled for example by amputation of healthy limbs or by taking actions to become paraplegic, blind or deaf (First, 2005; First and Fisher, 2011; Giummarra et al., 2012). This desire is accompanied by intense feelings of inappropriateness concerning the current non-disabled body configuration, described as “overcompleteness.” Since individuals with BID experience the apparently healthy and functionally intact bodies or body parts as not belonging, the disturbed body representations might qualitatively differ from the distortions in the context of the other disorders.

Comparing the age of onset of the disorders, BID stands out with a typical begin already in early to middle childhood at an age around 7 years (Blom et al., 2012; Brugger et al., 2016). Eating disorders usually develop during puberty or early adulthood with some evidence that AN starts slightly earlier than BN (Herpertz et al., 2019). An onset in later adulthood is less typical across eating disorders but most likely for BED (Stice et al., 2021). A large-scale epidemiological study with 10,123 adolescents aged between 13 and 18 years revealed median ages of onset of 12.3 (AN), 12.4 (BN) and 12.6 (BED; Swanson et al., 2011). The mean age of onset of BDD in adolescent samples is around the age of 12 to 13 years, with around 10% of the individuals experiencing a pre-pubertal onset (Albertini and Phillips, 1999; Rautio et al., 2022). For MD, the age of onset appears to be around the age of 19 (only retrospective studies available), sometimes evolving from anorectic symptoms (Olivardia et al., 2000). The later age of onset compared to BDD of the non-MD form and AN appears a bit surprising, given the similarities between the disorders. However, the known bias in retrospective studies estimating the age of onset to be higher than it actually was might have influenced these estimates.

Regarding the course of the disorders, BN and BED are rather variable inter- and intra-individually and reveal higher rates of (spontaneous and treatment supported) remission rates (World Health Organization, 2018) than the other disorders mentioned. The experienced overcompleteness in BID, as well as the course of AN, BDD and MD usually are chronic untreated, and even with psychological treatment, still most of the patients do not reach remission (First and Fisher, 2011; Hartmann et al., 2013; Krebs et al., 2017; Waldorf et al., 2019a; Underwood and Olivardia, 2022). For BID the situation is even worse as there are currently no effective (non-invasive) treatments leading to a remission or cure of BID (Chakraborty et al., 2021), despite several suggestions (e.g., Ramachandran and McGeoch, 2007; Lenggenhager et al., 2014).

### 4.2 Body schema distortions across the disorders

Compared to studies on the body image, less research has focused on distortions of the body schema across eating disorders, BDD, MD and BID.

Meneguzzo et al. (2023) report distortions in the body schema in individuals with versus without AN, even after weight recovery (i.e., atypical AN after treatment), as indicated by results from an image-word matching task. These results can be interpreted with regard to the interplay between body schema and body image, or – as suggested by the study authors - in the context of linguistic embodiment, as an integration of different sensory sources, motor imagery (i.e., body schema) and cognitive processes is needed for task processing. Other studies used a door like aperture task, measuring the ratio of shoulder rotation/aperture width as an indicator of the body schema in individuals with and without AN. The results reveal a body schema distortion in the clinical groups, which moved through the aperture as if they had a wider body than their actual body (Keizer et al., 2013; Beckmann et al., 2021). For AN, these findings already resulted in treatment approaches including components of body-scaled action, which reveal higher improvement rates for distortions in body representations compared to treatment as usual (Keizer et al., 2019).

For BDD, no study has yet focused on the body schema. Several studies used paradigms that are primarily intended to assess the body image, yet also activate the body schema. For example, studies used facial stimuli – presented with or without emotional expressions, or upright versus inverted – to investigate gazing behavior via eye-tracking (Grocholewski et al., 2012) or visual processing abnormalities via neuroimaging (Feusner et al., 2010). The tasks therefore primarily capture the body image, although indirect body schema influences are possible, since emotion recognition probably involves facial mimicry (Ponari et al., 2012) and inverted faces might induce mental rotation (Valentine and Bruce, 1988). Conversely, however, the tasks do not allow specific conclusions to be drawn about potential body schema distortions in BDD. In contrast to BDD of the non-MD type, typical MD behaviors (e.g., excessive weight-lifting) appear more closely related to the body schema. However, to our knowledge, no study has yet investigated body schema in muscle dysmorphia using, for example, action-oriented behavioral tasks similar to the aperture tasks in AN studies mentioned above.

BID is associated with specific neurological and behavioral characteristics. For example, individuals with BID show structural and functional changes in a distributed cortical network related to body representation, specifically, including sensorimotor areas, and parietal areas as well as the premotor cortex (Saetta et al., 2020, 2022). The use of a paradigm for mental rotation of body parts showed no evidence of a possible distortion of the body schema in BID (Stone et al., 2019). However, there are first indications that motor signals are differently processed in BID in the right paracentral lobule (Gandola et al., 2021). Moreover, as a behavioral characteristic, BID is very often accompanied by pretending behavior, that is the habitual simulation of the desired body state by using crutches or wheelchairs, or binding up the affected limb (First and Fisher, 2011; Giummarra et al., 2011; Blom et al., 2012). There is still relatively little known about the duration and frequency of pretending behavior in individuals with BID. However, case reports suggest that a certain degree of heterogeneity can be assumed (e.g., Kasten, 2009; Giummarra et al., 2011). Which behavior is expressed probably depends on the desired body configuration, that is subjects with the *amputation variant* of BID (Saetta et al., 2022) will mainly pretend by binding up the affected limb and using crutches, whereas the *paralysis variant* (Giummarra et al., 2012) will mainly use wheelchairs. We suggest that different forms of pretending are carried out to modify different kind of body related information. For example, the primary aim of using a wheelchair could be to simulate motor aspects of immobility (affecting the body schema), whereas the aim of tying up a body part could be either to modify the shape of the body (affecting the body image) or motor aspects of immobility of the limb (affecting the body schema) or both. Interestingly, a recent study demonstrated a correlation of neurological and behavioral markers of BID (Saetta et al., 2020). Unfortunately, the correlational nature of the link between pretending and body representation does not allow inferences on causality. One possibility is, that a distorted body representation in BID causes pretending behavior. However, an alternative possibility is, that pretending behavior (e.g., as a reaction to specific triggering events like encounters with severely physically ill and disabled individuals, see Obernolte et al., 2015) might cause a distorted body schema. Simulating a disabled body state severely limits and modifies the experience of movement of the body or body parts (Burin et al., 2017). In line with this, research on the effect of limb immobilization with healthy individuals has shown that temporary immobilization, such as through cast or splint, can lead to alterations in body representations of the immobilized body part. This has been shown for different factettes of body representations underlying body ownership, sensorimotor functioning, motor simulation and motor control (Toussaint and Meugnot, 2013; Burin et al., 2017; Debarnot et al., 2018; Scotto et al., 2020). Moreover, there are also first indications that limb immobilization might lead to changes in the brain (Huber et al., 2006; Langer et al., 2012). Future experimental studies are needed to clarify the causal role of pretending and distorted body representations in BID.

### 4.3 Body image distortions across the disorders

In eating disorders, many studies focus on the long-term visual cognitive-affective body image, often targeted with self-report questionnaires about one’s appearance. This research shows high body dissatisfaction and a high relevance of the body weight and shape for self-evaluation (for AN Glashouwer et al., 2019; across EDs Prnjak et al., 2022). Regarding the perceptual body image, individuals with AN show larger overestimation of their body sizes and body parts based on visual information (for a review, Gardner and Brown, 2014). A recent multi-center study on typical as well as atypical AN and weight bias, assessed with questionnaires and an experimental task with visual body stimuli, reported similar weight biases in individuals with and without (atypical) AN but higher body dissatisfaction (i.e., two aspects of the cognitive-affective body image) and a more pronounced overestimation of the own body (i.e., an aspect of the perceptual body image) in the clinical groups, especially in the individuals with atypical AN (Behrens et al., 2021). The authors discuss the role of cognitive-affective body image judgments as possibly more impactful compared to distortions in the perceptual body image. However, it appears challenging to disentangle the respective influences of specific distortions.

Other studies in AN have focused on other exteroceptive sensory sources, such as tactile information. For example, individuals with AN overestimate the size of certain body parts based on visual as well as tactile sensory information (Keizer et al., 2011). Similarly to the findings on body schema distortions in AN, these distortions of the perceptual body image even persist after successful weight rehabilitation (Engel and Keizer, 2017). In addition to studies revealing distortions in body image attributed to individual sensory sources, there is also evidence suggesting that multisensory integration is impaired in eating disorders. For example, studies using the rubber hand illusion showed that the influence of proprioceptive signals during the multisensory integration of proprioceptive und visual information is reduced in AN compared to healthy controls (Eshkevari et al., 2012; Zopf et al., 2016). Interestingly, the influence of touch is comparable in individuals with and without AN in the rubber hand illusion (Zopf et al., 2016) as well as in full body illusions, in which the synchronous visuo-tactile stimulation is used to induce embodiment of a virtual body (Keizer et al., 2016; Provenzano et al., 2020). These studies thus point to a weakened proprioceptive compared to exteroceptive influence in multisensory integration in AN. A study on the influence of auditory-driven signals on body weight perception in individuals with and without AN also revealed a weakened auditory influence in multisensory integration, speaking against an overreliance on exteroceptive information (Tajadura-Jiménez et al., 2022).

Besides exteroception, interoceptive abilities have been linked to the subjective experience of emotion (Paulus and Stein, 2006) and the cognitive-affective body image in particular (e.g., Duschek et al., 2015; Badoud and Tsakiris, 2017). Several authors propose a disturbance in the interoceptive system as a central mechanism of pathology in AN (e.g., Pollatos et al., 2008; Kerr et al., 2016; Jacquemot and Park, 2020). For example, patients with AN display significantly decreased interoceptive sensitivity as assessed by a heartrate perception task (Pollatos et al., 2008) which is evident even in recovered patients (Fischer et al., 2016). The insular plays a central role in the integration and representation of interoceptive signals (Craig, 2002) and AN symptoms have been associated with abnormal visceral interoceptive activity in the insula (Kerr et al., 2016). While the role of interoception is predominantly explored in AN, deficits in interoception have been observed across other types of eating disorders (BN and BED) and across several interoceptive modalities, such as gastric, pain, and cardiac interoception (Martin et al., 2019). However, the causal role of interoceptive body representations in eating disorders is not exactly clear. For example, in a recent longitudinal study with a non-clinical sample interoceptive accuracy did not predict body image dissatisfaction over a period of 8 weeks (Drew et al., 2020). Therefore, further prospective longitudinal studies with clinical populations as well as experimental psychopathology studies are needed to clarify this important question.

Similar to research in the field of eating disorders, most BDD studies focused on the cognitive-affective body image derived from visual information. Several studies targeted interactions between disorder specific behaviors involving visual information and effects on the cognitive-affective body image. For example, BDD-like gazing at facial photographs negatively affects attractiveness ratings, with different effects depending on the gazing duration and the type of stimuli, own versus other face (Möllmann et al., 2019, 2020b). Another study revealed a higher degree of negative affect, especially of sadness and anger, after body exposure in individuals with BDD compared to individuals with major depression and mentally healthy individuals (Kollei and Martin, 2014). Some studies on the perceptual body image in BDD investigated attentional biases during free viewing paradigms and reported a negative bias towards least liked areas of own and others face (Grocholewski et al., 2012; Toh et al., 2017). Different from AN, studies on body size estimations have not yet been published for BDD.

For MD, the research focus is similar. Individuals with MD show a negatively biased cognitive-affective body image. Regarding aspects of the visual perceptual body image, they show a positive bias towards hyper-muscular bodies of others versus a negative bias towards one’s own body, as assessed with eye-tracking and a mirror setup (Waldorf et al., 2019b) or an own body avatar presented in virtual-reality (Porras-Garcia et al., 2020). Other aspects of the perceptual body image, most typically body size estimations, have not yet been investigated in MD. For example, a recent systematic review on body image, muscle dysmorphia and eating disorders reported only two studies including a clinical group of individuals with MD across all body image facets, and these studies did not assess perceptual body image (Prnjak et al., 2022). As individuals with MD experience their bodies as too lean (contrary to individuals with AN), investigating body size estimations in this clinical group might be an interesting future path.

Other exteroceptive sensory sources have neither yet been targeted in BDD nor in MD research, although for example BDD behaviors, such as checking certain body parts, are closely related to other sensory information. Regarding interoception, one study reported a negative association between BDD symptoms and cardiac interoceptive accuracy in a group with low BDD symptoms but they did not find a significant association in the high BDD symptom group (Kunstman et al., 2016). In line with these findings, a recent longitudinal survey-based study revealed a similar association between MD symptoms and interoceptive sensibility, both cross-sectionally and longitudinally (Grunewald et al., 2023). Studies on multisensory integration in tasks assessing the perceptual body image in BDD and MD are rare. One study used the rubber hand illusion. Individuals with BDD and mentally healthy individuals did not show significantly different experiences of illusion strength (Kaplan et al., 2014), different from results on the rubber hand illusion effect in AN, and thus not indicating deficits in multisensory integration. However, the authors reported significant positive correlations between the illusion strength and body dissatisfaction ratings, indicating an interplay between the perceptual and cognitive-affective body image.

In the amputation variant of BID, several studies point to altered uni- and multisensory processing of the affected and unaffected limb that are probably linked to the perceptual and cognitive-affective body image. For example, there are indications for impaired spatial–temporal integration of tactile stimuli (Aoyama et al., 2012) as well as structural and functional changes in parietal areas (Saetta et al., 2020, 2022). The parietal cortex is associated with multisensory integration and the phenomenal components of the bodily self as revealed by direct electrical brain stimulation. Stimulating the superior parietal lobule in particular has been shown to result in illusory distortions of body size and the sensation that a body part is absent (Dary et al., 2023). Besides altered exteroceptive processing, individuals with BID also show altered interoceptive processing. For example, pain and pain anticipation (Brang et al., 2008; Romano et al., 2015) as well as disgust responses to violations of the body envelop are differently processed (Bottini et al., 2015). There are also first indications, that interoceptive awareness as assessed via self-report, is reduced in BID patients (Capodici et al., 2023). Together, these observations fit well to the observation of BID-related structural alterations in the insula, an area that is known to play an important role in the representation of interoceptive signals (McGeoch et al., 2011; Hilti et al., 2013).

## 5 Discussion

We introduced a conceptual framework on body representations with two interconnected models, the structural and the process model and explored if this framework is useful in improving our understanding of body representation distortions in mental disorders. We aimed to address inconsistencies in terminology and conceptualizations in the literature across different disciplines and shaped the potential transdiagnostic role body representation may contribute to understanding psychopathology.

We believe this conceptual work to be the only transdiagnostic framework of body representation so far which facilitates to structure and compare findings across research areas. The specific role of body representations for the selected mental disorders (BDD, MD, BID or eating disorders) is not always clear and it would be beneficial for intervention optimization if we knew certain aspects of body representations were a risk factor (and also causally related to the disorder development) or rather a feature of the disorder. We are motivated by the striking clinical observation that certain mental disorders, which are accompanied by distortions in the way the own body is perceived and represented, are severe and - despite treatments - quite persistent. These observations raise the question whether the severeness and chronicity of such disorders might be connected to the distortions in body representation. Other authors have already raised this question for a specific disorder, namely anorexia nervosa (Glashouwer et al., 2019). With the current review, however, we contribute three new aspects:

1. We proposed a conceptual framework on body representations allowing an *interdisciplinary perspective*, as it integrates concepts and terminology from different disciplines. The framework helps to tackle problems identified in previous research on body representations in the context of mental disorders (e.g., heterogeneity in terminology and concepts across and within disciplines; lack of experimental research), it facilitates the integration of previous research and also enables to derive future research directions (see also Section 5.2 Future directions).
2. To improve the understanding of body representations in the context of (mental) disorder development and maintenance, it appeared important to also take a *developmental psychopathology perspective on body representations* – irrespective of (mental) disorders - i.e. we reviewed research findings on the (normative) development of body representations across the lifespan.
3. We have extended the perspective of focusing on one specific mental disorder to a *transdiagnostic perspective on body representations* by comparing certain disorders that share the feature of distorted body representations but can be described as either more proximal or distal to each other regarding the disorder symptoms (eating disorders, body dysmorphic disorder including muscle dysmorphia, and body integrity dysphoria).

### 5.1 Synthesis and conclusions

Key differences in our model (compared to other available models) are that we (a) distinguish the three orthogonal dimensions of body representations and therefore provide a structuring system of categories, (b) distinguish body schema from body image, as these different functions may also differentially relate to disorders or conditions (e.g., as causal risk factor versus correlate), and (c) emphasize the sensory source more strongly in the first place (via a separate dimension) to then differentiate along the sensory input.

With the focus on the structural model in the current review, we may draw several conclusions about the amount of research activity to date. We identified certain major areas both in basic research and in relation to clinical conditions. Significantly more studies have addressed the body image than the body schema. And significantly more studies have focused on (certain) exteroceptive than interoceptive information. We believe that this is due to not only the possibly higher perceived relevance, but also with the higher complexity associated with investigating the other aspects, especially for research in children. The studies that we have reviewed actually indicate that this research may be expanded. For example regarding the different functions of body representation, we found evidence for developmental effects, indicating certain sensitive phases potentially associated with an increased risk for developing distorted body representations and thus maybe even for the development of mental disorders. For body schema, for example, (at least) two phases appeared relevant: unusual body-related experiences at a very young age, such as care routines after pre-term birth, can affect body schema development. Further, the growth spurt during puberty demands major adaptations in existing body representations due to the fast changes in body morphology. These findings indicate that it might be worthwhile to investigate the body schema more intensively. Due to the absence of longitudinal studies, we might so far only hypothesize that the early single effects of unusual body-related experiences during infancy could initiate developmental cascades. The accumulating effects may unfold only during puberty, as another sensitive phase, and only lead to permanent changes in the developmental result at this point. For adult AN and weight-restored AN as well as for BID, body schema distortions have been shown. Especially the findings on the persistence of these distortions even after treatment (also present for the perceptual body image) in AN suggest that these aspects may not yet be sufficiently targeted in the respective treatments, while potentially playing a role for relapse.

A similar imbalance in research activity was observed with regard to sensory sources. There are significantly more studies on exteroceptive sources (esp. visual) compared to interoception and proprioception. At the same time, the findings on interoception for distorted body representations and the clinical conditions indicate that the relevance of this source might currently be still underestimated. For example, although interoceptive accuracy was found to be associated to disordered eating in young people and reduced in (adult) AN, there are no or only single studies on the relevance of interoceptive information for BDD and MD in the transdiagnostic comparison. Furthermore, there are no experimental studies on body size estimations in BDD and MD, despite the known abnormalities in the AN.

### 5.2 Future research directions

To improve the understanding of the transdiagnostic role of body representations in the context of body-related (mental) disorders and to identify relevant new research questions, it seems reasonable to first depict the current state of research with systematic reviews and meta-analyzes. The structural model might be helpful in stimulating this kind of knowledge accumulation, as it allows both, a theory-driven and empirically driven approach to identify relevant and/or under-researched aspects of body representations (i.e., cells in the three-dimensional model) for any disorders or conditions. Similarly, it can be used to identify which aspects of body representations are already targeted in existing treatments. Based on our initial overview in the current article, several possible research gaps emerged, which may be substantiated in *a-priori* designed studies (e.g., the role of interoception across several disorders).

The second part of our conceptual framework - the dynamic process model - suggests different pathways of information processing and allows testing these pathways more closely in the formation, maintenance, or modification of distorted body representations. This might be especially useful from the developmental psychopathology perspective, if one aims to systematically investigate the “risk-factor status” (p. 341; Kraemer et al., 1997) of a body representation. For example, the *formation* of distorted body representations might be mainly driven by distorted (or absent) sensory input underlying dynamic short-term body representations (i.e., representations about how the own body currently is). If these unregular experiences take place during potential sensitive developmental phases, they may have the potential to initiate cascade effects spilling over to and stabilizing in long-term body representations (i.e., representations about how the own body usually is; see pathway 1 in the process model depicted in part b of Figure 1). This is because according to the process model, long-term representations themselves are constructed by accumulating short-term information about the own body. As mentioned above, atypical sensory stimulation due to premature birth (Butti et al., 2020) or the lack of visual input during the first months of life (Röder et al., 2004) might have profound effects in the development of the body schema and perceptual body image, respectively. In contrast, the *maintenance* of distorted body representations might be mainly driven by two additional pathways depicted in the process model. The first pathway is independent from the actual output and relies on the interplay between short-term and long-term representations (see pathway 2 in the process model). Once established, distortions can be imported from long-term memory and thus affect the way the body is dynamically represented as information from long-term memory is typically combined with the actual sensory input in working memory (e.g., for AN, Gadsby, 2017). As a consequence, precisely those now distorted short-term representations are used to further stabilize the distorted long-term representations. Another pathway possibly involved in the maintenance of distorted body representations is more dependent on the actual output (see pathway 3 in the process model). If distortions in body representations result in changes in body-related behaviors, as for example pretending behavior in BID or camouflaging in BDD, these outputs in themselves create newly distorted input that may further stabilize the distorted representations. The process model thus provides first testable hypotheses about how (distorted) body representations develop and how they are maintained. To clarify causality, future experimental psychopathology research is needed. Such research might for example test, whether experimentally manipulating sensory input and/or behavioral output has an impact on psychopathology. Finally, the process model can also be used for developing ideas regarding the *modification* of distorted body representations. This is especially relevant if one wants to proceed investigating a specific body representation, which has ideally already been identified as a risk factor. Consequently, the questions arise as to whether (1) this specific body representation can be modified at all and (2) the modifications lead to relevant changes in the disorder pathology (Kraemer et al., 1997). If so, these findings can directly inform translational research regarding prevention and treatment approaches.

## Author contributions

AM: conception, writing–original draft, and writing–review and editing. NH: conception, writing–original draft, writing–review and editing, resources, and supervision. AH: conception, writing–original draft, writing–review and editing, and supervision. All authors contributed to the article and approved the submitted version.
